# Developmental Pathways of *Psammotermes hybostoma* (Isoptera: Rhinotermitidae): Old Pseudergates Make up a New Sterile Caste

**DOI:** 10.1371/journal.pone.0044527

**Published:** 2012-09-17

**Authors:** Thomas Bourguignon, Jan Šobotník, David Sillam-Dussès, Pavel Jiroš, Robert Hanus, Yves Roisin, Toru Miura

**Affiliations:** 1 Graduate School of Environmental Science, Hokkaido University, Sapporo, Japan; 2 Evolutionary Biology and Ecology, Université Libre de Bruxelles, Brussels, Belgium; 3 Institute of Organic Chemistry and Biochemistry, Prague, Czech Republic; 4 Czech University of Life Sciences, Faculty of Forestry and Wood Sciences, Prague, Czech Republic; 5 Laboratoire Écologie et Évolution, Université Pierre et Marie Curie, Paris, France; 6 IRD, UMR 211 BIOEMCO, IBIOS, Bondy, France; 7 Laboratoire d’Ethologie Expérimentale et Comparée, Université Paris 13, Villetaneuse, France; University of Otago, New Zealand

## Abstract

**Background:**

Ergonomic efficiency is in termites maximized by task partitioning among specialized castes. The isopteran caste systems can be classified as either (i) linear, when tasks are performed by pluripotent immatures (pseudergates), retaining the ability to develop into winged imagoes or (ii) bifurcated, with the presence of a true worker caste, which diverges early and permanently from the sexual (nymph/alate) line.

**Principal Findings:**

Here, we report on the ontogenetic potentialities of the highly polymorphic sand termite *Psammotermes hybostoma*. Beside numerous pluripotent pseudergates, constituting the main work force, some larger non-feeding apterous immatures, also occur. These individuals are unable to proceed to the winged imago stage, but store large amounts of fat and also give rise to large soldiers. Soldiers therefore originate from a wide range of apterous instars, consequently being highly polymorphic.

**Conclusions:**

The caste system of *P. hybostoma* is essentially linear, as in other basal Rhinotermitidae, but is distinguished by the late bifurcation leading to large apterous immatures. Because these large worker-like individuals deviate late and do not perform worker tasks, they cannot be considered homologous to the true workers of Termitidae and advanced Rhinotermitidae, but they provide a novel example of the evolution of sterile immatures in termites.

## Introduction

In social insects, division of labour between nestmates implies that some individuals remain sterile and help a few relatives in order to enhance the number of fertile siblings. Released from their reproductive duties, these helper individuals develop a distinct morphology and behaviour, which can differ extraordinarily from the breeding phenotype. This specialization of individuals and their distribution between castes is the cornerstone of eusociality [1].

**Figure 1:**
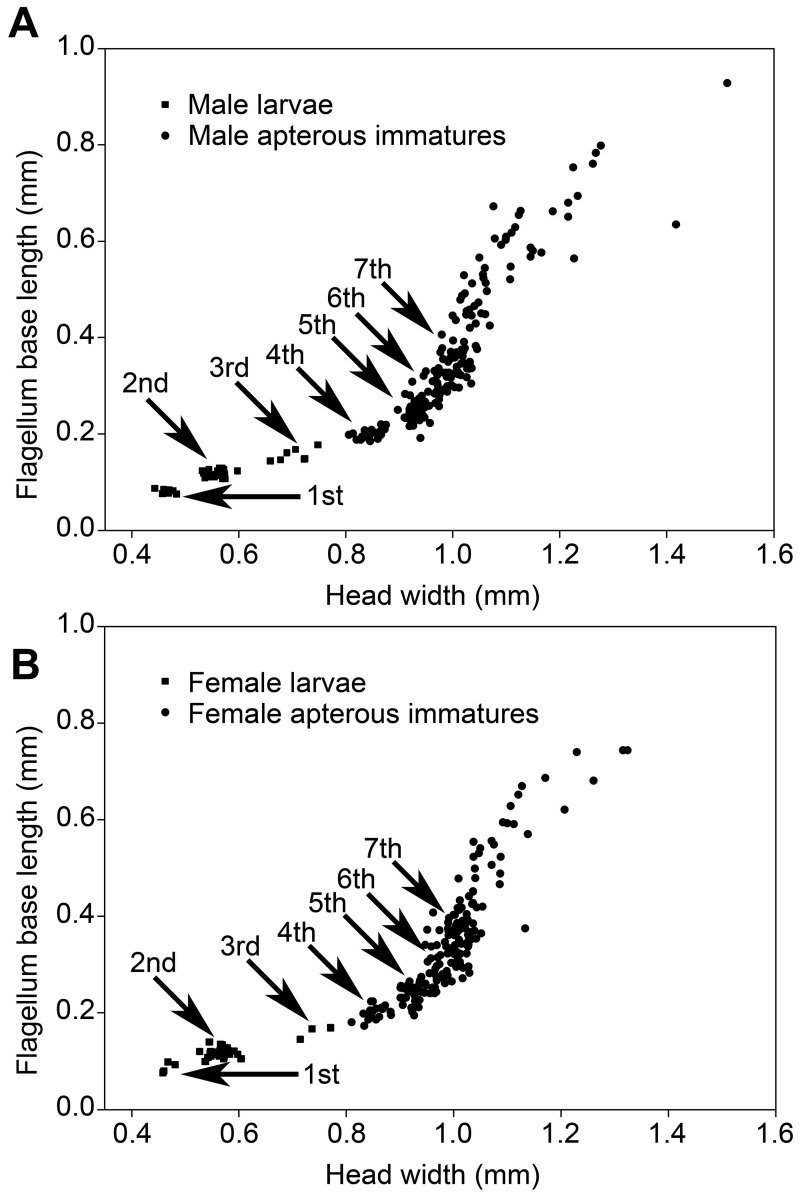
Head width versus flagellum base length of larvae and apterous immatures of *Psammotermes hybostoma*. Males (A) and females (B) from colony P3-1. Arrows point to the successive immature instars.

**Figure 2:**
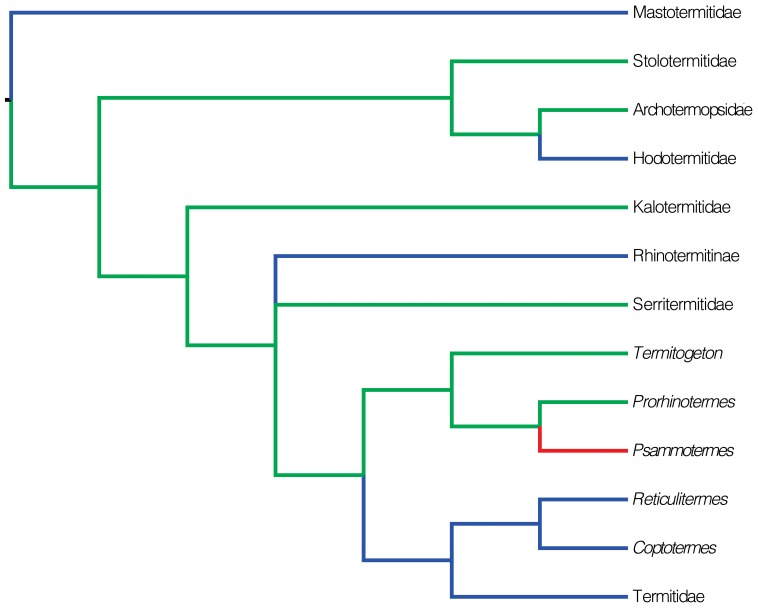
Phylogeny of termites, redrawn from the Maximum Likelihood analysis of Legendre et al. (2008). Nodes with bootstrap support below 50% were merged. *Psammotermes* was included in the phylogeny according to its retrieved position in Lo et al. (2004) and Inward et al. (2007). Tree shows the presumed evolution of pseudergates (in green), true workers (in blue) and the yet unknown *Psammotermes* (in red).

Termites represent one of the most diversified taxa of social insects, characterized by a hemimetabolous development. The typical development of hemimetabolous insects starts with eggs hatching into nymphs, which after a series of successive instars develop into imagoes. However, because termites are eusocial, some individuals step aside from the straight egg-to-imago pathway to produce distinct castes, such as soldiers. Soldiers are sterile, non-moulting individuals possessing a heavily sclerotized head capsule, endowed with specialized defensive features such as powerful biting or snapping mandibles, a thickened, plug-like forehead, or devices for squirting or rubbing a toxic secretion (Fig. 1). Soldiers represent an Isoptera synapomorphy, although they have been secondarily lost in a few groups of Termitidae [2]. In *Psammotermes*, soldiers are equipped with long and powerful biting mandibles able to wound opponents and a frontal gland filled with toxic and irritating compounds [3]. In some termite lineages, tasks such as food collection, construction or brood care are performed by immatures keeping the potentiality to proceed to the imago stage [2], [4]–[7]. These worker-like individuals are called pseudergates *sensu lato* [2], [4]–[7]. This linear developmental model is typical of basal termite lineages, i.e. Kalotermitidae, Archotermopsidae, Stolotermitidae (*sensu* [8]), Serritermitidae and the rhinotermitid genera *Prorhinotermes* and *Termitogeton* [7], [9]–[13] (Fig. 2). On the contrary, many termite taxa possess a caste of true workers, which deviate from the imaginal pathway at an early developmental stage and permanently forgo the potentiality to develop into winged imagoes. Such a development is termed bifurcated, and is characteristic of the Mastotermitidae, Hodotermitidae, some Rhinotermitidae (Rhinotermitinae, *Coptotermes*, *Heterotermes* and *Reticulitermes*) and all Termitidae [7], [14]–[19] (Fig. 2).

Whether termite ancestors had pseudergates *sensu lato* or true workers has been thoroughly debated [20]–[26]. The most likely scenario is that termite ancestors lived in small colonies sheltered in and feeding on a single piece of wood. Tasks were done by pseudergates, and all colony members (except soldiers) were able to develop into winged imagoes when food reserves dwindled. A true worker caste then evolved at least three times independently: once in Mastotermitidae, once at the origin of Hodotermitidae and at least once within a clade comprising Serritermitidae, Rhinotermitidae and Termitidae. The latter clade is particularly interesting because it includes several branches revealing intermediate characteristics, which prefigure the evolution of the true worker caste. In *Glossotermes oculatus* (Serritermitidae), the development is linear but the work is sex-partitioned; all tasks are performed by male pseudergates whereas females appear only shortly before the nuptial flights to proceed directly to winged imagoes [13]. In *Prorhinotermes inopinatus*, pseudergates can go out of their wood shelter piece to forage, behaviour previously considered specific of true workers [27]. On the other hand, two lineages of advanced termites possess true workers: the Rhinotermitinae and a clade comprising *Reticulitermes*, *Heterotermes*, *Coptotermes* and the Termitidae. Investigating the caste developmental systems of other related taxa might therefore reveal further unexpected patterns and allow a better understanding of the true worker caste origin. Unfortunately, the internal phylogeny of the [Serritermitidae + Rhinotermitidae + Termitidae] clade remains imperfectly resolved. The genus *Psammotermes* constitutes one of the basal lineages of this clade, but its affinities are uncertain: it has no close relatives, but some studies suggest that it might be the sister group of *Prorhinotermes* [28], [29]. *Psammotermes* is one of the few termites specialized on arid environments, tolerating extremely dry conditions [30]. Despite its phylogenetic position, its peculiar biology, and pest status [30], very few studies have attempted to improve the basic knowledge of *Psammotermes.* Here, in the continuity of the recent characterization of *Psammotermes hybostoma* trail-following pheromone, sex-pairing pheromone, and soldier defensive secretion [3], [31], we investigate its caste developmental system. Two previous studies emphasized the enormous polymorphism of *Psammotermes* soldiers and apterous immatures [32], [33], but provided only superficial insights into the developmental scheme.

## Results

### Terminology

**Figure 3:**
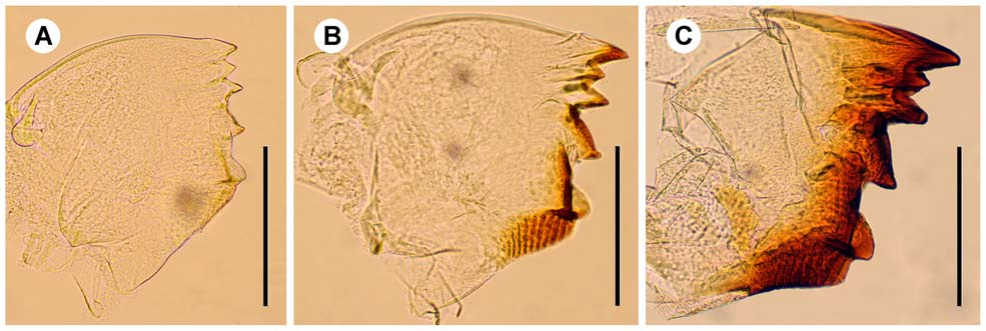
Left mandible of apterous immatures of the (a) second, (b) third and (c) fourth instars. Note the slight sclerotisation in the third instar. Scale bars, 0.2 mm.

We follow the terminology defined by [7] and [13]. In short:

Soldiers are characterized by their sclerotized head capsule and their elongated mandibles (Fig. 1).
Presoldiers are white, unsclerotized individuals with elongated mandibles. This is an inactive, transitional instar preceding the soldier stage.
Apterous immatures are whitish to yellowish individuals, devoid of wing rudiments and with gnawing mandibles. The youngest, presumably inactive instars are called larvae: they possess unsclerotized mandibles, and their digestive tract does not contain wood. Older apterous immatures appear as active feeders, as they possess sclerotized mandibles and their digestive tract is often filled with food (Fig. 1).
Neotenics are secondary reproductives, which reproduce without reaching the alate stage. They can be recognized by the buff color of their head and tip of abdomen.
Nymphs are whitish individuals with wing buds, on the developmental pathway to the alate stage (Fig. 3).
Alates are the winged imagoes.

### Apterous Immatures

**Figure 4:**
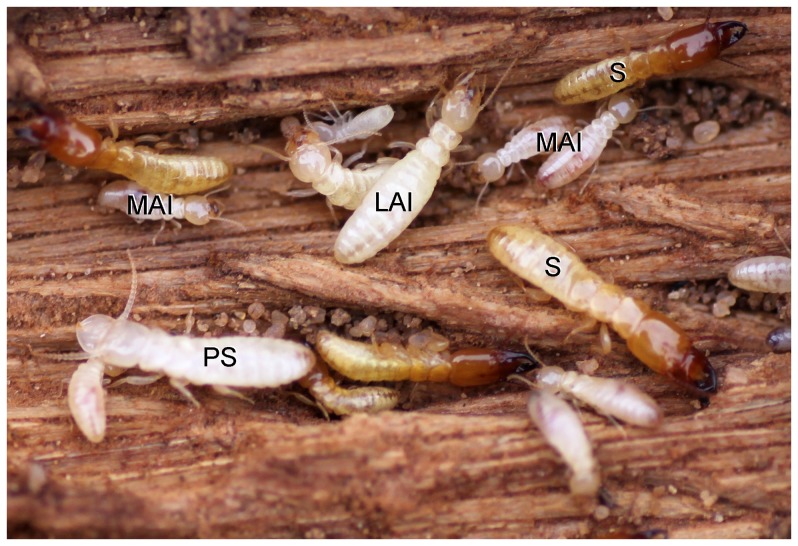
Live individuals of *Psammotermes hybostoma* in the field, after opening of a dead palm tree trunk. LAI, late apterous immatures; MAI, medium apterous immatures; PS, presoldier; S, soldiers.

Most of the 11 studied colonies included only a fraction of the apterous immature instars, generally lacking the early and/or the late instars. Larvae were only found in two colonies. They included the first three instars (Fig. 1). Third instar larvae have slightly sclerotized mandibles (Fig. 3), but their digestive tract is not filled with wood, suggesting that they are fed by nestmates and have not started working yet. The fourth instar has highly sclerotized mandibles (Fig. 3) and a digestive tract filled with wood, indicating that this is the first active wood-feeding apterous immature instar. The fourth instar is followed by a series of additional apterous immature instars increasing in size, making the oldest instars gigantic (Fig. 1). The fifth to seventh instars can be identified using morphometric data, but the number of subsequent instars is more uncertain, although over four can reasonably be assumed. Apterous immature instars do not only differ in size, but also by their appearance: the older individuals (hereafter referred as late apterous immatures) all have sharp mandibles, a digestive tract devoid of wood (like that of larvae or presoldiers), and high amounts of fat body; while the younger non-larval instars (hereafter referred as medium apterous immatures) often have their mandibles worn out, their digestive tract brownish, filled with wood, and comparatively little fat body (Fig. 4). The amount of triacylglycerols (TAGs), the dominant energy reserve of insects, is indeed more than 5 times higher in late immatures compared to medium ones (0.8% vs. 0.15%). Late apterous immatures were scarce, representing always less than one percent of the colony population while medium apterous immatures were very abundant, making the majority of the sampled individuals. The difference between medium and late apterous immature instars is not gradual but abrupt, occurring when individuals are about the size of nymphs.

**Figure 5:**
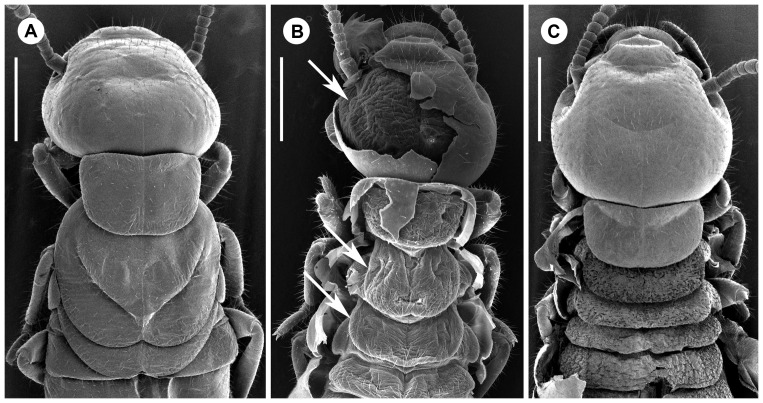
Scanning electron microscopy pictures. (a) Dorsal view of the thorax of a nymph. (b) Dorsal view on a pseudergate approaching the nymphal moult, with old cuticle partially peeled off. Arrows mark compound eye (laterally on the head) and wing pads on the thorax. (c) Dorsal view of a pseudergate approaching another pseudergate instar, with old cuticle peeled off from mesothorax, metathorax and abdomen. Scale bars, 0.5 mm.

We found three medium apterous immatures moulting into nymphs in colony P3-1, as evidenced by the presence of wing pads and eyes below the outer cuticle (Fig. 5B), both features being absent during pseudergate-pseudergate moults (Fig. 5C). This observation clearly shows that the development of *P. hybostoma* is linear and that apterous immatures are pseudergates *sensu lato*. Because moulting individuals have their cuticle slightly inflated, we could not precisely determine the pseudergate instars moulting into nymphs, but only inferred that they were within the size range of the 6^th^ to 8^th^ apterous immature instar. Whether only one or several of these instars can moult into nymphs is not known. Nymphs are of the size of the 7^th^ instar and present little polymorphism, which excludes the possibility that large late apterous immatures retain the ability to proceed into nymphs and alates. Therefore by the 9^th^ instar on, apterous immatures turn to late apterous immature morphs and probably lose the potential to moult into nymphs. We examined the mandibles of 131 moulting apterous immatures from colony P3-1 but found that all of them were moulting into the next apterous immature instar, suggesting that the presoldier moult is a rare event, at least in the season we carried out our sampling.

We tested for the presence of sexual size dimorphism for the first four apterous immature instars independently but never found any significant difference (Table 1). Apterous immature sex ratio is egalitarian in all colonies (Table 2).

**Table 1:** Mean value of measurements of castes tested for sexual dimorphism.

	Colony P2-1				Colony P3-1				Colony P4-1				Colony P6-1				Colony P6-2			
	n	HW	PW	FL	n	HW	PW	FL	n	HW	PW	FL	n	HW	PW	FL	n	HW	PW	FL
Larvae 1 ♂					8	0.465	0.317	0.081												
							**													
Larvae 1 ♀					4	0.467	0.334	0.087												
Larvae 2 ♂					25	0.560	0.368	0.118	9	0.581	0.375	0.116								
Larvae 2 ♀					29	0.565	0.375	0.118	15	0.580	0.377	0.117								
Larvae 3 ♂					8	0.704	0.443	0.156	27	0.742	0.467	0.165								
Larvae 3 ♀					3	0.740	0.466	0.161	40	0.741	0.470	0.163								
Immatures 1 ♂					18	0.846	0.521	0.200	11	0.897	0.559	0.214								
Immatures 1 ♀					21	0.852	0.523	0.200	17	0.907	0.565	0.220								
Neotenics ♂	6	1.046	0.759	0.531																
Neotenics ♀	14	1.041	0.741	0.515																
Nymph ♂					5	1.020	0.688	0.398					12	1.080	0.758	0.452	21	1.075	0.740	0.464
							*							**	**			*	**	
Nymph ♀					10	1.032	0.709	0.426					10	1.047	0.725	0.430	9	1.102	0.776	0.463
Alate ♂	13	1.036	0.825	0.470									43	1.080	0.850	0.543				
			**											***	**					
Alate ♀	8	1.051	0.850	0.478									15	1.102	0.880	0.547				

Footnote: Significant differences between males and females were investigated with *t*-test:

* p<0.05;

** p<0.01;

*** p<0.001. HW, head width (mm); PW, pronotum width (mm); FL, flagellum base length (mm); n, number of individuals measured.

**Table 2:** Male and female proportions among instars.

Colony:	P2-1		P3-1		P4-1		P4-2		P4-3		P5-1		P6-1		P6-2	
	♂	♀	♂	♀	♂	♀	♂	♀	♂	♀	♂	♀	♂	♀	♂	♀
Larvae 1			8	4												
Larvae 2			25	29	9	15										
Larvae 3			7	3	27	40										
Helpers			191	191	11	17	18	23					51	35	42	61
Soldiers			96	77			34	30	27	32	40	21*				
Nymphs			5	10									12	10	21	9*
Alates	15	11											45	18***		
Neotenics	8	17	2	3							4	5				

Footnote: Significant departure from 1∶1 ratio were investigated with Chi-square tests:

* p<0.05;

** p<0.01;

*** p<0.001. Helpers included all apterous immatures but larvae.

### Neotenics

Neotenics were found in three colonies. No other functional reproductives were found. They were all apterous, suggesting that they originated from apterous immatures. Neotenics can be distinguished by their darker, more pigmented abdominal tip. Many neotenics also have a brownish abdomen, thorax and head. Some female neotenics were slightly physogastric. We did not find any sign of sexual dimorphism and all individuals had 16-segmented antennae. All neotenics were of the size of the 8^th^ apterous immature except for two individuals of colony P3-1, which were of the size of the 9^th^ or 10^th^ apterous immature, suggesting that several successive apterous immature instars have the potentiality to develop in neotenics (Fig. 1). The sex ratio was not significantly biased, although this might result from the limited number of neotenics we found (Table 2).

### Nymphs and Alates

Nymphs are peculiar by the shape of the wing buds, which are connected to each other up to their tips (Fig. 5A). Altogether, we observed about 50 alates and 50 nymphs. Nymphs appear to form a single morph, although antennal size variation of nymphs suggests that they originate from several instars of pseudergates. Nymphs and alates generally have 16 antennal segments, sometimes 15, which are often imperfectly separated in nymphs, making the counting ambiguous in many specimens. In nymphs and alates, males were significantly smaller than females in all colonies but one, in which male nymphs were found significantly larger than female nymphs (Table 1). For both nymphs and alates, the sex ratio was biased towards males in one colony but not in others (Table 2).

### Soldiers

**Figure 6:**
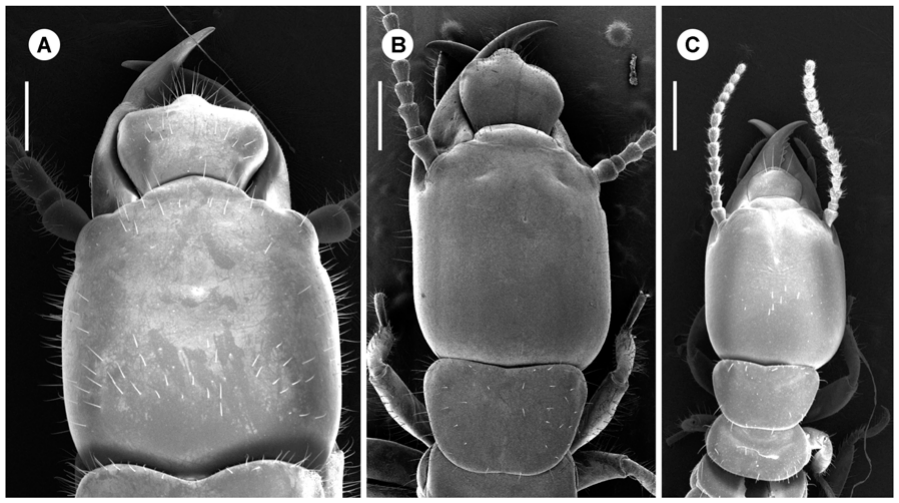
Soldiers of three different instars of *Psammotermes hybostoma*, at the same scale. Scale bars, 0.5 mm.

**Figure 7:**
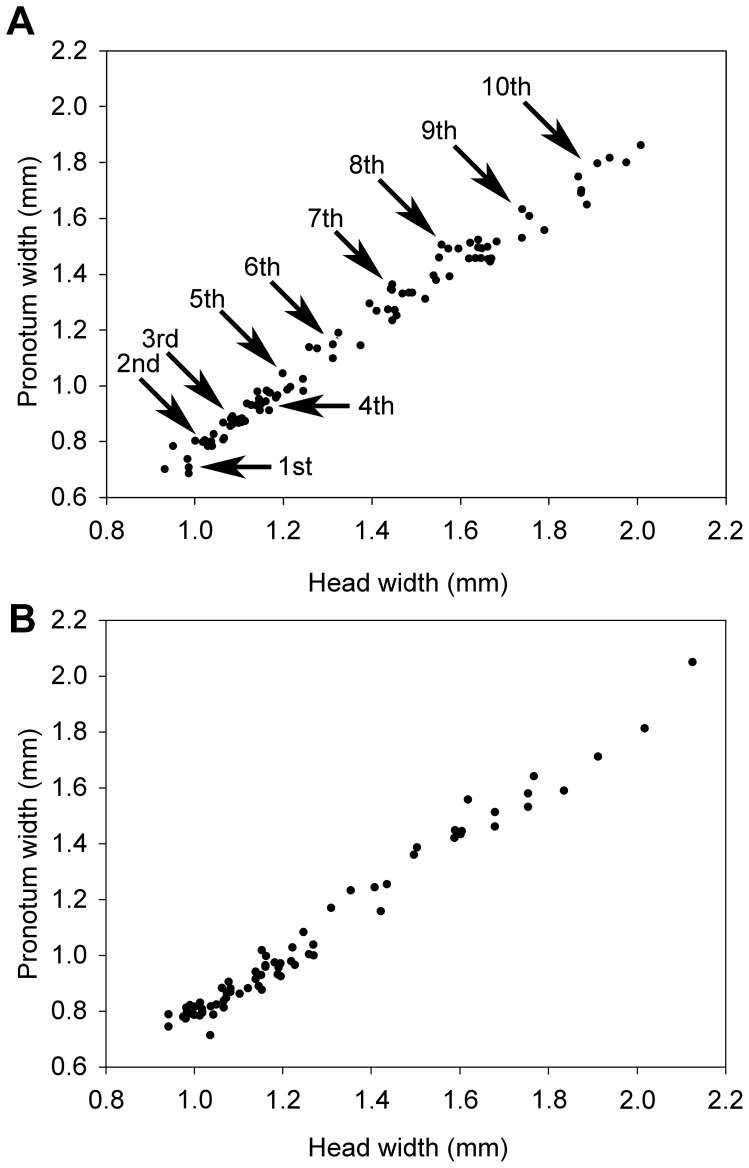
Head width versus pronotum width of male (A) and female (B) soldiers from colony P3-1. Arrows point to each soldier instar originating from successive apterous immature instars. Note that the first instar corresponds to the smallest soldiers we found in our colonies, but we cannot exclude the existence of younger soldier instars, especially in incipient colonies.

**Figure 8:**
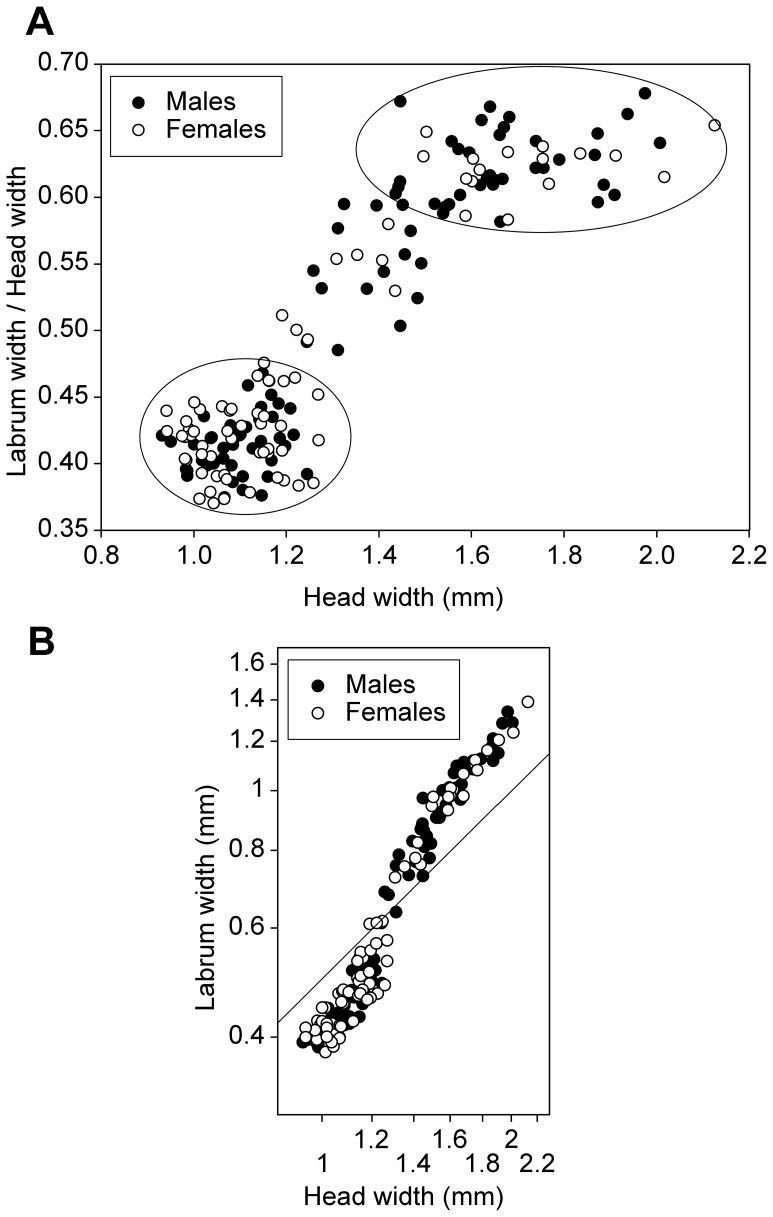
Soldiers from colony P3-1. (A) Head width versus proportional size of labrum (labrum width/head width), ellipses show the small and large soldier instars, having proportionally small and large labrum, respectively. (B) Labrum width versus head width on a logarithmic scale, the line is isometric (slope = 1).

Soldiers were highly variable in size, as well as in number of antennal segments which varied between 12 and 16 (Figs. 6, 7). As for apterous immatures, most colonies only contained a fraction of the soldier morphs, with the larger ones being scarcely represented. We particularly focused our morphometric study on colony P3-1 which was the only one to include the full range of soldier sizes. Morphometric analyses revealed the presence of 10 morphs clearly distinguishable in males (Fig. 7). One larger soldier was also found in females, suggesting the existence of at least one more morph (Fig. 7). Both male and female soldiers occur along the full size range. A slight allometry can be observed along the size gradient in soldiers, with a proportionally larger labrum (with a well-developed labral gland; Šobotník, unpublished) (Figs. 6 and 8). Males were significantly more abundant than females in one colony, but no biased sex ratio was found in two other colonies. We were not able to test for sexual size dimorphism because of imperfect separation of soldier instars.

## Discussion

**Figure 9:**
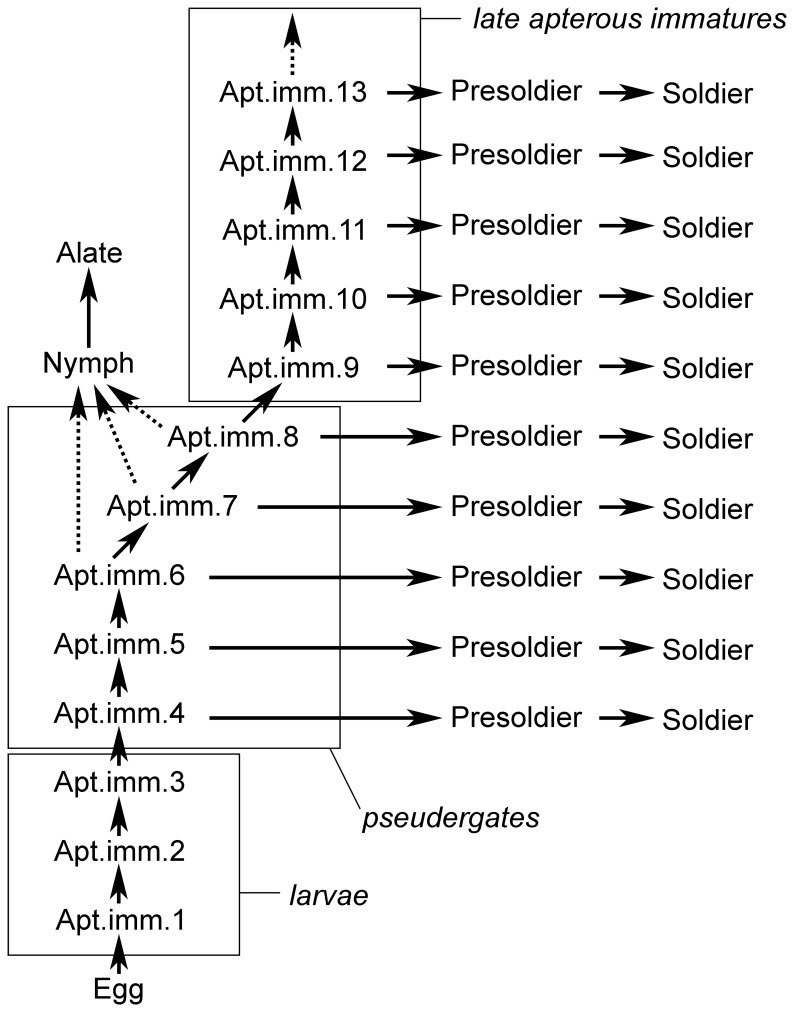
Proposed developmental scheme of the caste pathway of *Psammotermes hybostoma*. Arrows indicate the moults, deduced from the observation of moulting individuals or inferred from the polymorphism. Dashed arrows indicate the inferred moult into nymphs, the precise instars able to carry it out being hypothetical. Here, we proposed that the apterous immature (Apt. imm.) instar 8 represents the decision point, but we cannot completely exclude that it actually occurs slightly later. Neotenics are not represented because their precise origins were not investigated.

*Psammotermes* is subterranean, forming huge colonies which can reach over 10^5^ individuals with the nest located in the ground [30]. We collected the material only from wood and we did not find any nest, what explains the absence of eggs and the scarcity of larvae and reproductives in our samples. However, we collected enough specimens of each category to schematize the developmental pathway (Fig. 9).

Dependent larvae of *P. hybostoma* can easily be separated into three successive instars, followed by a series of apterous immatures. The wing development takes place through a single nymphal instar originating from apterous immatures. The latter can thus be called pseudergates. Such a development (proneometaboly *sensu* [34]) also occurs in the archotermopsid *Hodotermopsis* [35]–[37], in the rhinotermitid genera *Prorhinotermes* [11], [38] and *Termitogeton* [12], and in the serritermitid *Glossotermes* [13]. The sex ratio is generally egalitarian in all castes and sexual dimorphism is at most very small. One exception came from one colony whose male nymphs were larger than females. This apparent discrepancy might possibly be explained by a differential origin of nymphs, i.e. the females appear generally slightly larger than males, but male nymphs might originate from older instars in some colonies, being then larger than females. Low or absent sex specialization coupled with a low or absent sexual dimorphism is characteristic of linear developmental models with pseudergates as work force, such as those of, e.g., *Prorhinotermes* and *Neotermes* [11], [39].

In this study, we did not investigate the precise internal mechanisms responsible for individual moults. Although the exact mechanism controlling termite caste differentiation is still not completely understood, juvenile hormone titre is known to play a key role, especially during soldier differentiation [40]–[42]. Additionally, the combined action of juvenile hormone and ecdysone participates to the maturation of neotenic reproductives [43], [44]. Similar mechanisms must be behind the complex caste system of *P. hybostoma* but still have to be investigated.

A close look at the full range of soldiers reveals the presence of two main morphs composed by the small and the large individuals, which differ in their allometric relationships between the head and the labrum width. The small soldiers have a proportionally small labrum and thereby a small labral gland (Fig. 8), a large frontal gland approximately reaching the fourth abdominal segment and wispy legs, while large soldiers have a large labral gland, a small frontal gland confined into the head [3] and robust legs. The allometric relationship concerns defensive traits, suggesting that there is a selection upon soldiers to be either of the small or of the large type, as evidenced by the relatively low number of intermediates between these two extremes (see Fig. 8). The small and the large soldiers might be specialized towards different predators (e.g. ants vs. centipedes), as they rely upon different weapons. Although the polymorphism within soldier allometric categories might be adaptive, it might also be a non-adaptive by-product of the very special developmental pattern of *P. hybostoma*.

A remarkable feature of *P. hybostoma* is that pseudergates can continue moulting far beyond the pre-nymphal stage, giving rise to another category of apterous immatures. These immatures are unable to become imagoes, as evidenced by their huge body size dramatically exceeding that of medium apterous immatures, nymphs and alates. Medium and late apterous immatures differ not only in size, but also in anatomy: late apterous immatures have larger fat reserves, unworn mandibles and wood-free digestive tracts. Based on these observations, we expect that medium and late apterous immature instars fulfil distinct functions within the colony. Their proportion in the colony is also different, medium instars forming the majority of the population, whereas late ones are scarce. While medium instars perform the working tasks, late ones can be seen as an innovation of *P. hybostoma*. Clément [32] postulated that late apterous immatures have no other functions than proceeding into large soldiers. The formation of large soldiers is probably an important function of late apterous immatures, but this does not necessarily rule out a role for late apterous immatures on their own. One of their main characteristics is their whitish appearance resulting from their reserve of fat bodies, more than five times larger compared to medium apterous immatures. Here, we suggest that these huge fat reserves might be used to produce metabolic water (see [45]), a particularly important resource for a species vulnerable to desiccation and living in arid environment.

Although scarce and of uncertain function, the late apterous immatures are an obvious singularity of *P. hybostoma*, interesting from the developmental and evolutionary viewpoints. From around the 9th instar on, these late immatures reach a size preventing them from resuming alate development. A comparable situation was also observed in *Prorhinotermes simplex* in which small groups of pseudergates, isolated from the remainder of the colony, were able to survive for a surprisingly long time, moulting up to the 16th instar and becoming considerably larger than any pseudergate living in the functional colony (Hanus & Šobotník, personal observation). Such late instars of pseudergates might potentially be selected if they provide some benefit to the colony. In the desert-living *P. hybostoma*, water storage (as lipids can be converted into water, see [45]) would be a likely function. It is tempting to compare the late apterous instars of *P. hybostoma* and the true workers occurring in several Rhinotermitidae and in all Termitidae, as both constitute permanent, eusocial castes. True workers probably originated from pseudergates which, in view of low chances of reaching the alate stage and high potential benefits of helping, shifted their ontogenetic bifurcation from the alate pathway earlier and earlier [46]. In *P. hybostoma*, the late apterous immatures are unlikely to constitute an incipient true worker caste because they fulfil other functions than the common helping tasks still performed by totipotent pseudergates. However, they illustrate how easily a new permanent caste may arise from termite pseudergates.

The developmental pathway of *P. hybostoma* is atypical in many respects: (i) its enormous polymorphism; (ii) the combination of huge colonies probably numbering hundreds of thousands of individuals with the presence of pseudergates *sensu lato*; and (iii) the development of a permanent caste of late apterous immatures, whose tentative function is to produce metabolic water when needed, and to give rise to huge soldiers. Without considering this latter caste, which only comprises scarce individuals, the caste system of *Psammotermes* is very similar to the one of *Prorhinotermes* [11], probably its sister genus as suggested by recent phylogenetic studies [28], [29]. Available evidence shows that a great adaptive diversification in life styles occurred along basal lineages of the [Rhinotermitidae + Serritermitidae + Termitidae] clade. With the present description of the developmental pathways of *P. hybostoma*, the major goal for future studies is to figure out the evolution of the Rhinotermitidae social systems, what could only be achieved through computing a more robust phylogenetic tree.

## Materials and Methods

### Termite Collecting and Preservation

*Psammotermes hybostoma* colonies were collected from tamarisk wood at the periphery of Sohag, Egypt, and from tamarisk or date palm trunks in the Western desert (for the localities see [3]). Termites were found in a system of plain galleries covered by a thick layer of faecal material mixed with sand, but were in general not encountered deeper in the tamarisk wood, whereas they fed on date palm trunks from inside. Specimens from 11 colonies were collected and fixed in FAA (formol:alcohol:acetic acid; 20∶75:5). We paid special attention to the collection of immature individuals approaching a moult, which can be recognized by their whitish appearance, and of large immatures and soldiers, which represent only a few percent of colony members (Fig. 4). Subsamples from each category were then randomly selected for anatomical, morphological, or biometric studies.

### Measurements

For all specimens studied biometrically, we took a series of pictures with an Olympus DP50 camera mounted on an Olympus SZH binocular microscope. Pictures were then analyzed with the software ImageJ 1.42q software (Wayne Rasband, National Institutes of Health, USA). Maximal width of head (HW) and pronotum (PW) was measured for all specimens. The base of the antennal flagellum is generally the part of the antenna that is the most affected by the growth at each moult in termites [16]. As segments are often ill-defined in this region, it is generally not possible to count precisely the number of antennal segments. Therefore, we chose to measure the distance between the junction of scapus and pedicel, and the junction of the ninth and eighth segment counted from the apex (FL). These two junctions were always well-marked and this distance was measured in a homologous way among all specimens, from the first instar larvae with 11 antennal segments to large apterous immatures with up to 17 antennal segments. We did not measure this distance for soldiers as the number of antennal segments was largely variable among instars and between both antennae of the same individuals. We chose to replace this measure by the width of the labrum (LW).

### Triacylglycerols (TAG) Content Analysis

Samples of 10 medium apterous immatures and 7 late apterous immatures from FAA were dried under stream of argon for 30 minutes and weighted. Bodies were transferred to a clean vial and covered with chloroform (approx. 150 µL), crushed with glass stick and ultrasonicated for 5 minutes. Whole extract was separated on the silicagel thin layer chromatography glass plate. Zone with TAG was scrapped and eluted with 2 mL of diethyl ether. Solution was transferred over small filter of wool to pre-weighted vial. Net weight of TAG was measured after drying under argon stream (15 min).

### Sex Determination

Sex determinations were carried out as explained in [13] and [16]. Overall, the gonads resemble those of *Termitogeton planus* [12]. Females were recognised by the presence of ovaries, spermatheca and colleterial glands, while males present testes and seminal vesicles.

### Outcome of Moults

Individuals approaching a moult are recognizable by their opaque white body. If fixed shortly before exuviation, the new cuticle can be seen through the old one and reveals the outcome of the approaching moult. In particular, the presence of bulging incipient wing pads in apterous immatures reveals that they are about to moult into nymphs (Fig. 5). After removing the old cuticle, the new cuticle can be visualized using scanning electron microscopy.
