# Correction: Developmental Pathways of *Psammotermes hybostoma* (Isoptera: Rhinotermitidae): Old Pseudergates Make up a New Sterile Caste

**DOI:** 10.1371/annotation/426a9f1f-f8dc-4f42-a929-adfdc3e117f9

**Published:** 2012-09-27

**Authors:** Thomas Bourguignon, Jan Šobotník, David Sillam-Dussès, Pavel Jiroš, Robert Hanus, Yves Roisin, Toru Miura

There was an error in the published Funding statement. The correct Funding is:

Financial support for this study was received from the Czech Science Foundation (project P506/10/1570; http://www.gacr.cz/); the Institute of Organic Chemistry and Biochemistry (project No. RVO: 61388963; http://www.uochb.cz); the Czech University of Life Sciences and the Ministry of Agriculture of the Czech Republic (project No. QH91097; http://www.czu.cz/en/); Japan Society for Promotion of Science (postdoctoral fellowship to TB, 22-00393; http://www.jsps.go.jp/english/); Grants-in-Aid for Young Scientists, the Ministry of Education, Culture, Sports, Science and Technology of Japan(project 21677001; http://www.jsps.go.jp/english/). The funders had no role in study design, data collection and analysis, decision to publish, or preparation of the manuscript. 

